# *Parabacteroides massiliensis* sp. nov., a new bacterium isolated from a fresh human stool specimen

**DOI:** 10.1016/j.nmni.2019.100602

**Published:** 2019-09-07

**Authors:** S. Bellali, C.I. Lo, S. Naud, M.D.M. Fonkou, N. Armstrong, D. Raoult, P.-E. Fournier, F. Fenollar

**Affiliations:** 1) Aix Marseille Université, IRD, AP-HM, MEФI, Marseille, France; 2) IHU-Méditerranée Infection, Marseille, France; 3) Aix Marseille Université, IRD, AP-HM, SSA, VITROME, Marseille, France

**Keywords:** Bacteria, culturomics, human gut, *Parabacteroides massiliensis*, taxono-genomics

## Abstract

*Parabacteroides massiliensis* sp. nov., strain Marseille-P2231^T^ (= CSURP2231 = DSM 101860) is a new species within the family *Tannerellaceae*. It was isolated from a stool specimen of a 25-year-old healthy woman. Its genome was 5 013 798 bp long with a 45.7 mol% G+C content. The closest species based on 16S rRNA sequence was *Parabacteroides merdae* strain JCM 9497^T^ with 98.19% sequence similarity. Considering phenotypic features and comparative genome studies, we proposed the strain Marseille-P2231^T^ as the type strain of *Parabacteroides massiliensis* sp. nov., a new species within the genus *Parabacteroides.*

## Introduction

Currently, the genus *Parabacteroides* includes eight valid species with standing in nomenclature [1]. Among them, *Parabacteroides distasonis*, *Parabacteroides goldsteinii* and *Parabacteroides merdae* previously belonged to the genus *Bacteroides* but were reclassified as members of the genus *Parabacteroides* since 2006 [2]. The species *Parabacteroides faecis* [3] and *Parabacteroides johnsonii* [4] (faeces) and *Parabacteroides gordonii* (blood) [5] were all isolated for the first time in humans. Culturomics is a concept developing different culture conditions to enlarge our knowledge of the human microbiota through the discovery of previously uncultured bacteria [6], [7], [8], [9]. Once it was isolated, we used a taxono-genomics approach including matrix-assisted laser desorption/ionization time-of-flight mass spectrometry (MALDI-TOF MS), phylogenetic analysis, main phenotypic description and genome sequencing, to describe this strain [10], [11]. Here we describe a new *Parabacteroides massiliensis* sp. nov., strain Marseille-P2231^T^ (= CSURP2231 = DSM 101860) according the concept of taxono-genomics.

## Isolation and growth conditions

**Fig. 1:**
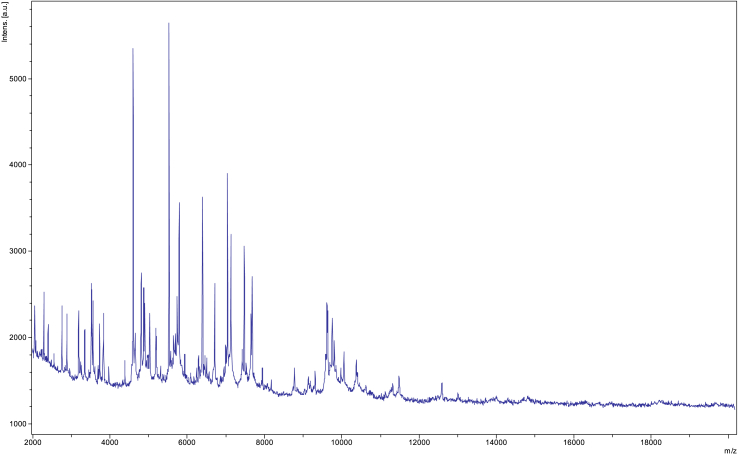
MALDI-TOF MS reference mass spectrum of *Parabacteroides massiliensis* sp. nov. Spectra from 12 individual colonies were compared and a reference spectrum was generated.

In 2017, we isolated from a fresh stool sample of a 25-year-old healthy woman an unidentified bacterial strain. Screening was performed using MALDI-TOF MS on a Microflex LT spectrometer (Bruker Daltonics, Bremen, Germany) as previously described [12]. The obtained spectra (Fig. 1) were imported into MALDI Biotyper 3.0 software (Bruker Daltonics) and analysed against the main spectra of the bacteria included in two databases (Bruker and the constantly updated MEPHI databases). The study was validated by the ethics committee of the IHU Méditerranée Infection under number 2016-010. Initial growth was obtained after 72 hours of culture in a Colombia agar enriched with 5% sheep's blood (bioMérieux, Marcy l’Etoile, France) in strict anaerobic conditions at 37°C and pH 7.5.

## Strain identification

**Fig. 2:**
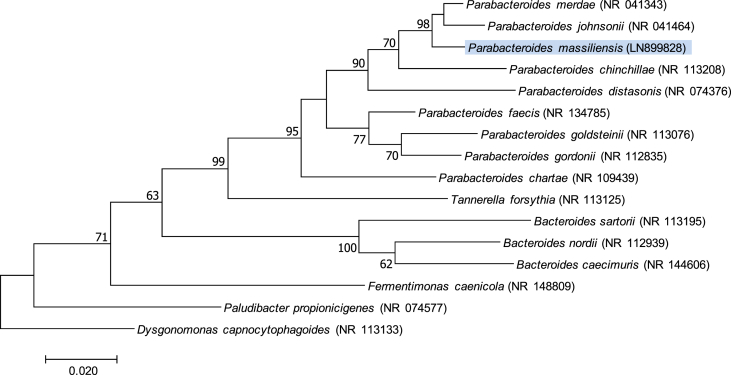
Phylogenetic tree showing the position of *Parabacteroides massiliensis* strain Marseille-P2231^T^ relative to other phylogenetically close neighbours. The respective GenBank accession numbers for 16S rRNA genes are indicated in parenthesis. Sequences were aligned using MUSCLE v3.8.31 with default parameters and phylogenetic inferences were obtained using the maximum likelihood method within MEGA 7 software. Numbers at the nodes are percentages of bootstrap values obtained by repeating the analysis 1000 times to generate a majority consensus tree. The scale bar indicates a 2% nucleotide sequence divergence.

The 16S rRNA gene was sequenced to classify this bacterium. Amplification was carried out using the primer pair fD1 and rP2 (Eurogentec, Angers, France) and sequencing using the Big Dye® Terminator v1.1 Cycle Sequencing Kit and ABI Prism 3130xl Genetic Analyzer capillary3500xLGenetic Analyzer capillary sequencer (Thermofisher, Saint-Aubin, France), as previously described [13]. The 16S rRNA nucleotide sequences were assembled and corrected using CodonCode Aligner software (http://www.codoncode.com). Strain Marseille-P2231^T^ exhibited a 98.19% sequence identity with *Parabacteroides merdae* strain JCM 9497^T^ (GenBank accession number NR_041343), the phylogenetically closest species with standing in nomenclature (Fig. 2). We consequently classify this strain as a member of a new species within the family *Tannerellaceae*, phylum Bacteroidetes.

## Phenotypic characteristics

**Fig. 3:**
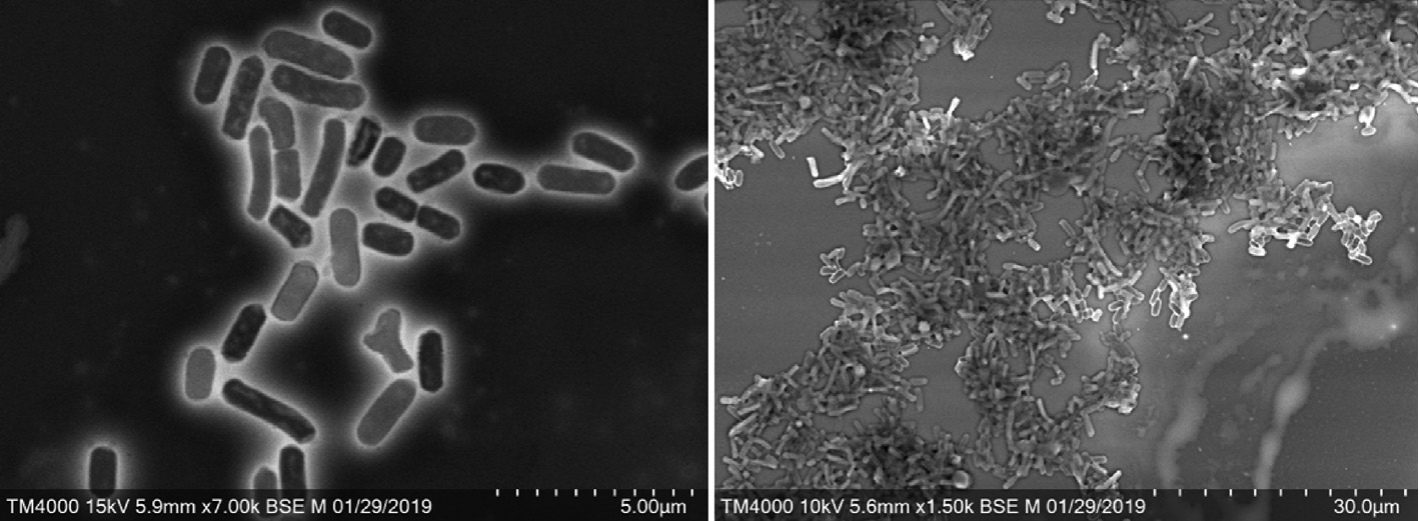
Scanning electron micrograph of *Parabacteroides massiliensis* strain Marseille-P2231^T^ using TM4000 microscope from HITACHI. Scale bar and acquisition settings are shown on the original micrograph.

Colonies were circular and smooth with a mean diameter of 1.2 mm. Bacterial cells were Gram-negative, rod-shaped, ranging in length from 1.27 to 2.46 μm and in width from 0.45 to 0.73 μm (Fig. 3). Strain Marseille-P2231^T^ showed catalase-negative and oxidase-negative activities. Main phenotypic properties of strain Marseille-P2231^T^ were studied by using the API 50 CH strips (Table 1), API ZYM strips (Table 2) and API 20A strips (Table 3). The main characteristics of strain Marseille-P2231^T^ are summarized on digitalized protologue (www.imedea.uib.es/dprotologue) under the number TA00985. The biochemical and phenotypic features of strain Marseille-P2231^T^ were compared with those of other close representative strains in the *Porphyromonadaceae* family (Table 4)

**Table 1:** Biochemical tests of *Parabacteroides massiliensis* (API 50 CH strips)

Tests	Results	Tests	Results
Control	–	Esculin	+
Glycerol	–	Salicin	+
Erythrol	–	d-cellobiose	+
d-arabinose	–	d-maltose	+
l-arabinose	–	d-lactose	+
d-ribose	–	d-melibiose	+
d-xylose	w	d-saccharose	+
l-xylose	+	d-trehalose	+
d-adonitol	–	Inulin	–
Methyl βd-xylopyranoside	+	d-melezitose	+
d-galactose	+	d-raffinose	w
d-glucose	+	Starch	w
d-fructose	+	Glycogen	–
d-mannose	+	Xylitol	–
l-sorbose	–	Gentibiose	w
l-rhammose	–	d-turanose	+
Dulcitol	–	d-lyxose	–
Inositol	–	d-tagatose	w
d-mannitol	w	d-fucose	–
d-sorbitol	–	l-fucose	–
Methyl αd-mannopyranoside	–	d-arabitol	–
Methyl αd-glucopyranoside	w	l-arabitol	–
*N*-acetylglucosamine	+	Potassium gluconate	–
Amygdalin	+	Potassium 2-ketogluconate	–
Arbutin	–	Potassium 5-ketogluconate	+

**+**, positive result; **−**, negative result; w, weakly positive.

**Table 2:** Biochemical tests of *Parabacteroides massiliensis* (API ZYM strips)

Tests	Results
Alkaline phosphatase	+
Esterase (C4)	–
Esterase Lipase (C8)	–
Lipase (C14)	–
Leucine arylamidase	+
Valine arylamidase	–
Cystine arylamidase	–
Trypsin	–
α-chymotrypsin	–
Acid phosphatase	–
Naphthol-AS-BI-phosphohydrolase	–
α-galactosidase	+
β-galactosidase	+
β-glucuronidase	+
α-glucosidase	–
β-glucosidase	–
*N*-acetyl- β-glucosaminidase	+
α-mannosidase	–
α-fucosidase	–

**+**, positive result; **−**, negative result.

**Table 3:** Biochemical tests of *Parabacteroides massiliensis* (API 20A strips)

Tests	Results
l-tryptophan	+
Urea	–
d-glucose	**+**
d-mannitol	+
d-lactose	+
d-saccharose	+
d-maltose	+
Salicin	+
d-xylose	+
l-arabinose	+
Gelatin (bovine origin)	+
Esculin ferric citrate	+
Glycerol	–
d-cellobiose	+
d-mannose	+
d-melezitose	+
d-raffinose	–
d-sorbitol	–
l-rhamnose	+
d-trehalose	+

**+**, positive result; **−**, negative result.

**Table 4:** Differential characteristics of 1, *Parabacteroides massiliensis* strain Marseille-P2231, compared with other closely related *Porphyromonadaceae* species: 2, *Parabacteroides merdae*[2]; 3, *Parabacteroides johnsonii*[4]; 4, *Parabacteroides gordonii*[5]; 5, *Parabacteroides faecis* strain 157^T^[3]; 6, *Parabacteroides chartae* NS31-3^T^[22]

Properties	1	2	3	4	5	6
Cell diameter (μm)	0.4–0.7	0.8–1.6	0.8	0.8	1.0	0.7–1.0
Oxygen requirement	−	−	−	−	−	−
Gram stain	−	−	−	−	−	−
Motility	−	−	−	−	−	−
Endospore formation	−	−	−	−	−	−
Acid phosphatase	−	NA	NA	NA	NA	+
Catalase	−	−	+	variable	+	−
Indole	−	−	−	−	−	−
Urease	−	−	−	−	−	−
Alkaline phosphatase	+	+	+	+	+	+
β-galactosidase	+	+	+	+	+	+
Mannose	+	+	+	+	+	+
Raffinose	w	+	+	+	+	+
Sucrose	+	+	+	+	+	+
Glucose	+	+	+	+	+	+
d-xylose	+	+	+	+	+	+
Maltose	+	+	+	+	+	+
Glycerol	−	−	−	−	−	−
Lactose	+	+	+	+	+	+
G+C content (mol%)	45.7	44.0	47.6	44.6	41.8	37.2
Habitat	Human stool	Human faeces	Human faeces	Human blood	Human faeces	Wastewater

+, positive result; −, negative result; w, weakly positive; NA, data not available.

Cellular fatty acid methyl ester analysis was performed by gas chromatography/mass spectrometry. Two samples were prepared with approximately 5 mg of bacterial biomass per tube harvested from several culture plates. Fatty acid methyl esters were prepared as described by Sasser [14]. Gas chromatography/mass spectrometry analyses were performed as described elsewhere [15]. The most abundant fatty acid by far was 12-methyl-tetradecanoic acid (43%), followed by 3-hydroxy15-methyl-hexadecanoic acid (19%) and hexadecanoic acid (10%). Several branched structures and specific 3-hydroxy fatty acids were described. Minor amounts of unsaturated and other saturated fatty acids were also detected (Table 5).

**Table 5:** Cellular fatty acid composition (%) of *Parabacteroides massiliensis* strain Marseille-P2231^T^

Fatty acids	Name	Mean relative % a
15:0 anteiso	12-methyl-Tetradecanoic acid	43.1 ± 1.1
17:0 3-OH iso	3-hydroxy-15-methyl-Hexadecanoic acid	18.5 ± 0.4
16:0	Hexadecanoic acid	9.5 ± 0.5
16:0 3-OH	3-hydroxy-Hexadecanoic acid	5.0 ± 0.2
15:0	Pentadecanoic acid	4.5 ± 0.3
15:0 iso	13-methyl-Tetradecanoic acid	3.5 ± 0.2
17:0 3-OH anteiso	3-hydroxy-14-methyl-Hexadecanoic acid	4.8 ± 0.8
18:2n6	9,12-Octadecadienoic acid	2.3 ± 0.1
5:0 iso	3-methyl-Butanoic acid	2.0 ± 0.2
18:1n9	9-Octadecenoic acid	1.9 ± 0.1
16:1n7	9-Hexadecenoic acid	1.1 ± 0.1
14:0	Tetradecanoic acid	TR
17:0 3-OH	3-hydroxy-Heptadecanoic acid	TR
17:0 anteiso	14-methyl-Hexadecanoic acid	TR
17:0 iso	15-methyl-Hexadecanoic acid	TR
14:0 iso	12-methyl-Tridecanoic acid	TR
18:0	Octadecanoic acid	TR
16:0 anteiso	13-methyl-Pentadecanoic acid	TR
13:0 iso	11-methyl-Dodecanoic acid	TR
17:0	Heptadecanoic acid	TR
13:0 anteiso	10-methyl-Dodecanoic acid	TR

a Mean peak area percentage; TR, trace amounts <1%.

## Genome sequencing

**Fig. 4:**
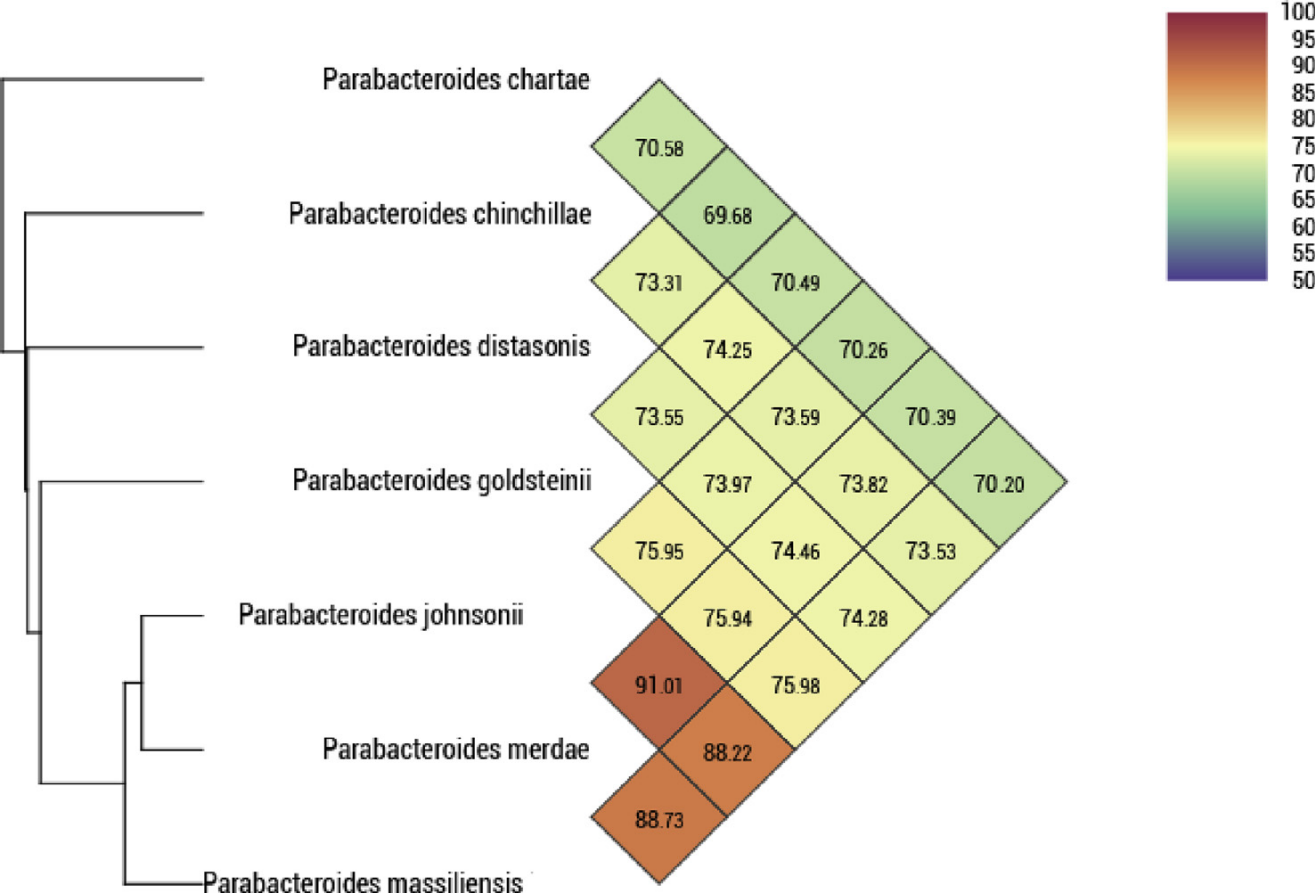
Heatmap generated with OrthoANI values calculated using the OAT software between *Parabacteroides massiliensis* and other closely related species with standing in nomenclature.

Genomic DNA was extracted using the EZ1 biorobot (Qiagen, Courtaboeuf, France) with the EZ1 DNA tissue kit and then sequenced using MiSeq technology (Illumina, San Diego, CA, USA) with the Nextera Mate Pair sample prep kit (Illumina), as previously described [16]. The assembly was performed with a pipeline incorporating different software (Velvet [17], Spades [18] and Soap Denovo [19]), and trimmed data (MiSeq and Trimmomatic [20] software) or untrimmed data (only MiSeq software). GapCloser was used to reduce assembly gaps. Scaffolds <800bp in length and scaffolds with a depth value <25% of the mean depth were removed. The best assembly was selected using different criteria (number of scaffolds, N50, number of N). The genome of strain Marseille-P2231^T^ is 5 013 798 bp long (23 scaffolds, 27 contigs, 762 401 N50) with a 45.7 mol% G+C content and contains 4 195 predicted genes. The degree of genomic similarity of Marseille-P2231^T^ with closely related species was estimated using the OrthoANI software [21]. Values among closely related species (Fig. 4) ranged from 70.20% between *Parabacteroides massiliensis* and *Parabacteroides chartae* to 91.01% between *P. merdae* and *P. johnsonii*. When the isolate was compared with these closely related species, values ranged from 70.20% with *P. chartae* to 88.73% with *P. merdae*.

## Conclusion

Strain Marseille-P2231^T^ exhibiting a 16S rRNA sequence divergence <98.7% and an OrthoANI value <95% with its phylogenetically closest species with standing in nomenclature, is consequently proposed as the type strain of the new species *Parabacteroides massiliensis* sp. nov.

### Description of *Parabacteroides massiliensis* sp. nov.

*Parabacteroides massiliensis* (mas.si.li.en'sis, L. fem. adj., *massiliensis*, ‘of Massilia’, the Latin name of Marseille, where this strain was isolated). Cells are obligate anaerobic, Gram-negative, non-motile and non-spore-forming. Catalase and oxidase activities are negative. Cells have a length of 1.27–2.46 μm and a width of 0.45–0.73 μm. Colonies grown at 37°C on 5% sheep-blood-enriched Columbia agar (bioMérieux), and were circular and smooth after 72 hours of incubation under anaerobic conditions. They had a mean diameter of 1.2 mm on agar. Strain Marseille-P2231 reacts positively with leucine arylamidase, alkaline phosphatase, α-galactosidase, β-galactosidase, β-glucuronidase, *N*-acetyl-β-d-glucosaminidase, d-glucose, d-fructose, d-mannose, esculin, salicin, lactose, melibiose, sucrose and potassium 5-ketogluconate. Negative reactions were observed with esterase, lipase, trypsin, acid phosphatase, naphthol-AS-BI-phosphohydrolase, β-glucosidase, α-mannosidase, α-fucosidase, glycerol, ribose, d-adonitol, rhammose, sorbitol, inulin, glycogen, xylitol, fucose, arabitol, arabitol and potassium 2-ketogluconate. The most abundant fatty acid by far was 12-methyl-tetradecanoic acid (43%) followed by 3-hydroxy 15-methyl-hexadecanoic acid (19%) and hexadecanoic acid (10%). The genome is 5 013 798 bp long and its G+C content is 45.7 mol%. Strain Marseille-P2231^T^, isolated from a fresh stool sample of a 26-year-old healthy woman, was deposited in the CSUR and DSMZ collections under accession numbers CSURP2231 and DSM 101860, respectively. The 16S rRNA and genome sequences are available in the GenBank database under accession numbers LN899828 and FTLH00000000, respectively.

### Nucleotide sequence accession number

The 16S rRNA gene and genome sequences were deposited in GenBank under accession number LN899828, and FTLH00000000, respectively.

### Deposit in culture collections

Strain Marseille-P2231^T^ or strain SN4^T^ was deposited in strain collection under number (= CSURP2231^T^ = DSM 101860).
